# Machine learning assisted real-time deformability cytometry of CD34+ cells allows to identify patients with myelodysplastic syndromes

**DOI:** 10.1038/s41598-022-04939-z

**Published:** 2022-01-18

**Authors:** Maik Herbig, Angela Jacobi, Manja Wobus, Heike Weidner, Anna Mies, Martin Kräter, Oliver Otto, Christian Thiede, Marie‑Theresa Weickert, Katharina S. Götze, Martina Rauner, Lorenz C. Hofbauer, Martin Bornhäuser, Jochen Guck, Marius Ader, Uwe Platzbecker, Ekaterina Balaian

**Affiliations:** 1 Biotechnology Center, Center for Molecular and Cellular Bioengineering, Technische Universität Dresden, Dresden, Germany; 2 Center for Regenerative Therapies Dresden (CRTD), Technische Universität Dresden, Dresden, Germany; 3 Max Planck Institute for the Science of Light & Max-Planck-Zentrum für Physik und Medizin, Erlangen, Germany; 4 Medical Department I, University Hospital Carl Gustav Carus Dresden, Dresden, Germany; 5 Medical Department III, University Hospital Carl Gustav Carus Dresden, Dresden, Germany; 6 German Cancer Consortium (DKTK), Partner Site Dresden and German Cancer Research Center (DKFZ), Heidelberg, Germany; 7 Zentrum für Innovationskompetenz: Humorale Immunreaktionen in Kardiovaskulären Erkrankungen, Universität Greifswald, Greifswald, Germany; 8 Department of Medicine III: Hematology and Oncology, School of Medicine, Klinikum Rechts Der Isar, Technical University of Munich, Munich, Germany; 9 Center for Healthy Aging, Dresden, Germany; 10 Department of Hematology, Cellular Therapy and Hemostaseology, Leipzig University Hospital, Leipzig, Germany

**Keywords:** Computational science, Diseases, Haematological diseases, Computer science, Characterization and analytical techniques, Imaging techniques

## Abstract

Diagnosis of myelodysplastic syndrome (MDS) mainly relies on a manual assessment of the peripheral blood and bone marrow cell morphology. The WHO guidelines suggest a visual screening of 200 to 500 cells which inevitably turns the assessor blind to rare cell populations and leads to low reproducibility. Moreover, the human eye is not suited to detect shifts of cellular properties of entire populations. Hence, quantitative image analysis could improve the accuracy and reproducibility of MDS diagnosis. We used real-time deformability cytometry (RT-DC) to measure bone marrow biopsy samples of MDS patients and age-matched healthy individuals. RT-DC is a high-throughput (1000 cells/s) imaging flow cytometer capable of recording morphological and mechanical properties of single cells. Properties of single cells were quantified using automated image analysis, and machine learning was employed to discover morpho-mechanical patterns in thousands of individual cells that allow to distinguish healthy vs. MDS samples. We found that distribution properties of cell sizes differ between healthy and MDS, with MDS showing a narrower distribution of cell sizes. Furthermore, we found a strong correlation between the mechanical properties of cells and the number of disease-determining mutations, inaccessible with current diagnostic approaches. Hence, machine-learning assisted RT-DC could be a promising tool to automate sample analysis to assist experts during diagnosis or provide a scalable solution for MDS diagnosis to regions lacking sufficient medical experts.

## Introduction

Hematopoiesis takes place in the bone marrow (BM) and is driven by hematopoietic stem cells (HSCs) which proliferate or differentiate along the blood cell lineages. At a certain stage of maturation, blood cells leave the BM and enter the blood circulation. Myelodysplastic syndromes (MDS) are malignant disorders, caused by genetic mutations in HSCs, resulting in impaired blood cell differentiation, and emergence of dysplastic cells in the BM and peripheral blood with propensity to progress into acute myeloid leukemia. However, the genetic defects and clonal evolution in MDS are not yet fully understood and do not provide the definitive delineation of MDS from reactive states and clonal hematopoiesis of indeterminate potential (CHIP)^1,2^. Therefore, the gold standard of MDS diagnostics currently still relies on a morphologic analysis of stained smears of cells from BM aspirates or biopsies. According to the recommendation of the World Health Organization (WHO), at least 100 erythroblasts, 100 granulocytic cells, and 30 megakaryocytes need to be evaluated in a BM smear to diagnose MDS^3^. Interestingly, these WHO guidelines suggest consideration of features such as cell size, but the assessment is purely visual. Such lack of quantification could lead to inconsistent results^4^. Furthermore, the visual assessment is time consuming and requires medical expertise which may not be available in each hospital. Therefore, a standardized and automated acquisition and quantification of the cell morphologies is desired.

**Figure 1:**
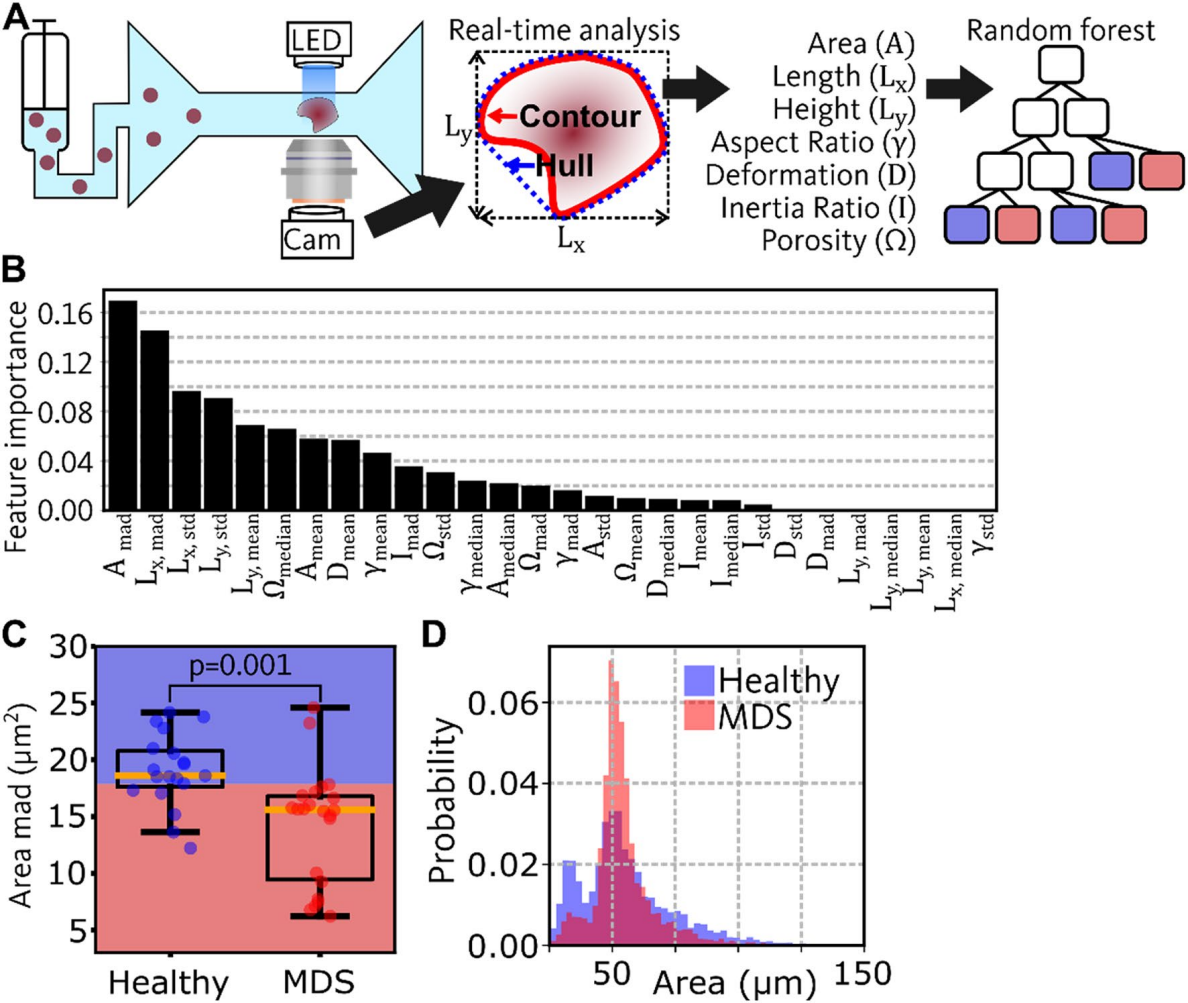
Detection of MDS using RT-DC and machine learning. (**A**) Sketch on the left shows the RT-DC setup. A syringe pump pushes suspended cells into a microfluidic chip which has a narrow constriction channel. Within the channel, cells are deformed and captured by a high-speed camera. Images are analyzed in real-time to obtain the contour (red), the convex hull (blue), the bounding box (dashed lines), and compute seven features. For each feature, the mean, median, standard deviation, and median absolute deviation are computed, resulting in 28 features describing the distributions. Sketch on the right illustrates a random forest model, which was trained to discriminate healthy and MDS, based on the 28 distribution features. (**B**) Barplot shows the feature importance values of a random forest model that was trained using 28 distribution features to discriminate healthy and MDS. (**C**) Boxplot shows the median absolute deviation (*mad*) of area for healthy and MDS samples. A random forest model was trained on that single feature to distinguish healthy and MDS and the resulting decision boundary is shown in the boxplot (blue corresponds to healthy and red to MDS). Boxes show the interquartile ranges ($IQR$), which are defined by the 25th percentile (${Q}_{1}$) and the 75th percentile (${Q}_{3}$): $IQR={Q}_{3}-{Q}_{1}$. Yellow lines in the boxes show the medians. Whiskers represent the range of the data (lower bound: ${Q}_{1}-1.5\cdot IQR$, upper bound: ${Q}_{3}+1.5\cdot IQR$). (**D**) Histograms show the distribution of area for representative measurements of healthy (blue) and MDS (red).

Real-time deformability cytometry (RT-DC) is an imaging flow-cytometry technique that performs image segmentation fully automated in real-time (see Fig. 1A)^5^. RT-DC records cell morphology, described by the outline of each cell (red contour in Fig. 1A), at rates up to 1000 cells/s, allowing for rare cell population detection and resolve even slight shifts of populations^6^. A syringe pump is employed to drive suspended cells into a microfluidic chip, where they are captured inside a narrow channel constriction slightly wider than the cell’s diameter. Since hydrodynamic forces in the channel deform the cell, the outline also contains information of the mechanical properties. Thus, RT-DC can broaden diagnostical parameters to cell mechanical properties, inherent markers of cell function^7^.

It is already known, that motility and cytoskeletal structure of HSCs show differences between the healthy state and MDS^8–12^. Furthermore, cell contractility could be altered during MDS due to a significantly increased expression of the interleukine-8 receptor (CXCR2)^13,14^. The regulation of molecules such as Rho GTPases and associated kinases, which are relevant for cell morphology, are affected in MDS^15^ and AML^16^. Hence, detection of HSCs with abnormal mechanical properties could be a promising diagnostic marker for MDS.

In the present study, we used RT-DC to capture the phenotype of BM-derived CD34^+^ HSCs from MDS patients and age-matched healthy donors. Seven features were extracted from the contour (red in Fig. 1A) of each cell and a random forest model was trained to distinguish between the healthy state and MDS. We used a model interpretation technique to identify features that are most discriminative between healthy and MDS. Further, we analyzed the correlation between HSC deformation and mutational status.

## Materials and methods

### Real-time deformability cytometry (RT-DC)

The central element of RT-DC is a PDMS (PDMS, SYLGARD 188, Dow Corning) based microfluidic chip with a narrow channel constriction (squared cross section, width = 20 µm). Sheath and cell suspension fluid are supplied to the chip using two syringe pumps (neMESYS 290 N, neMESYS) (Fig. 1A only shows one syringe pump) at a flow rate of 0.04 µl/s^5^. The measurement buffer for RT-DC (CellCarrier, Zellmechanik Dresden) is based on PBS which is complemented with 0.5% methylcellulose (w/w) and has a viscosity of 15 mPas (at 24 °C). The chip is placed on an inverted microscope (Zeiss Axiovert 200 M, Zeiss) equipped with a 40 × objective (NA = 0.75, Neofluar, Zeiss). The sheath fluid focuses the sample stream towards the channel constriction. Moving cells are captured in the channel at 3000 frames per second by a high-speed camera (EoSens CL, MC1362, Mikrotron) which triggers an LED (CBT-120, Luminus Devices) for illumination (2 µs light pulses). The cells are deformed due to hydrodynamic forces, which results in characteristic bullet-like cell-shapes, depending on their mechanical properties. Image data is sent to a PC via a full camera link frame grabber card (NI-1433, National Instruments). For data acquisition, the software ShapeIn (Zellmechanik Dresden) was employed. The images are analyzed in real-time to obtain the contour and compute seven features describing the morphology of each single cell:

- *Area (A)* By counting the number of pixels inside the convex hull of the contour (blue in Fig. 1A), the projected area of the cell is computed, which is a descriptor for cell size.
- *Length (L*_x_*) and height (L*_y_*)* A bounding box is fitted to the contour to determine length and height of the tracked object.
- *Aspect ratio (γ)* Based on L_x_ and L_y_, the aspect ratio is computed using $\gamma =\frac{{L}_{x}}{{L}_{y}}$.
- *Deformation (D)* Based on the area and perimeter (l) of the convex hull of the contour (blue in Fig. 1A), deformation is computed by $D=1-\frac{2\sqrt{\pi A}}{l}$. Deformation describes the deviation of the captured shape from a perfect circle.
- *Inertia Ratio (I)* The contour (red in Fig. 1A) describes the shape of an object. More elongated objects contain patches of area more distant from the centroid which can be quantified by the second moment of area. The second moment of area with respect to the x and y-direction is ${I}_{xx}={\iint }_{A}{y}^{2}dx dy$ and ${I}_{yy}={\iint }_{A}{x}^{2}dx dy$, respectively. To describe the relative elongation of an object, we introduce the inertia ratio as $I= \frac{{I}_{yy}}{{I}_{xx}}$.
- *Porosity (Ω)* By relating the area described by the convex hull (blue in Fig. 1A) and the original contour (red in Fig. 1A), a parameter is derived which expresses how irregularly shaped an object is: $\Omega =\frac{{A}_{hull}}{{A}_{contour}}$.

### Bone marrow sample preparation

BM samples from 19 healthy donors (median age at BM sampling 70.0 years [range, 47–87]; m/f = 6/13) and 22 patients (pts, median age at BM sampling 71.5 years [range, 43–87]; m/f = 12/10) were collected during total hip arthroplasty operations or at diagnostic BM puncture, respectively. Nine of these pts presented with lower risk MDS (low and intermediate-1 risk group according to International Prognostic Scoring System, IPSS), two pts had higher risk MDS (intermediate-2 and high according to IPSS), four pts had progressed to secondary acute myeloid leukemia with low proliferative activity (AML, 20–30% blasts), six pts exhibited myelodysplastic/myeloproliferative (MDS/MPN) overlap syndrome (including chronic myelomonocytic leukemia and refractory anemia with ring sideroblasts and thrombocytosis), and one pt had an overlap syndrome of MDS and aplastic anemia. The studies were approved by the Ethics committee at the Technical University Dresden and conducted in accordance with the Helsinki Declaration. Written informed consent was obtained from all patients.

MDS was diagnosed based on morphological analysis of BM smears by two independent hematologist experts.

BM was diluted in phosphate buffered saline (PBS), complemented with 2 mM EDTA and 0.5% human serum albumin. Mononuclear cells (MNCs) were separated by Ficoll-Paque (1.077 g/cm^3^, GE Healthcare) and washed twice using PBS. MNCs were enriched using a positive selection of CD34^+^ antigen using magnetic activated cell sorting (MACS) according to the manufacturer’s instructions (CD34 MicroBead Kit, human, Miltenyi Biotec). The purity of CD34^+^ enriched cells was above 95% which was verified by flow cytometric analysis (FACSCalibur analyzer, BD Biosciences). The resulting samples were cryopreserved using DMEM with 20% fetal bovine serum, 1% penicillin/streptomycin, and 10% DMSO. Samples were thawed in a 37 °C water bath, spun down and cells were resuspended in measurement buffer for RT-DC.

### Mutational analysis

Molecular alterations were evaluated for 10 MDS samples by targeted resequencing on a MiSEQ instrument (Illumina) using an amplicon assay (True Sight Myeloid Panel, Illumina) which covers 54 genes and gene hotspots related to myeloid neoplasms. A list of the tested mutations can be found in Supplementary Table 1.

### Random forest

Random forests consist of assemblies of decision trees that are trained on random subsets of the dataset^17^. Classification is performed using the majority vote of all trees. As each tree is trained on a random subset of the dataset, the remaining data can be used for validation and the resulting score is termed out-of-bag accuracy (oob accuracy).

An ideal feature for classification would allow to split up the entire dataset correctly into the existing classes. Less performant features only allow to guide a small portion of the data into the correct classes. Before a split, the data is usually mixed (impure) and after the split as pure as possible. Impurity is quantified using the Gini impurity index:

$$G=1-\sum_{i=1}^{{n}_{c}}{p}_{i}^{2}$$

with the number of classes ${n}_{c}$ and the fraction of events that belong to class $i$ ${p}_{i}$ (${p}_{i}=$ number of events of class $i$ divided by the total number of events). If a feature separates the data well, it decreases the Gini impurity index:

$$\Delta G={G}_{parent}-\left({G}_{Split1}+{G}_{Split2}\right)$$

Therefore, the $G$ can be used to quantify the importance of a feature for the overall classification performance. We used the Python implementation for random forest classifiers from the sklearn package (version = 0.20.0)^18^. This implementation allows customization of hyper parameters, including number of trees (n_estimators_), features to consider when spitting (n_features_), and maximum depth of each tree (n_depth_). For model training, we either used the default hyper parameters (defined for sklearn package version = 0.20.0^18^), or we optimized those hyper parameters individually by iterating the parameter and fixing the parameter, once a maximum of the oob accuracy was reached. For the dataset presented in this work, we found the following optimal values: n_estimators_ = 10, n_features_ = 4, n_depth_ = 4.

### Statistical analysis

Analyses were performed using Python 3.5.6. The median absolute deviation (*mad*) is the average (median) distance between data points and the median and was computed using:

$$mad=median(abs(x-median\left(x\right))/c$$

Here, $x$ are the measured values, $abs$ indicates the absolute function, and $c=0.6745$. The two-sided t-test was performed using the Python package SciPy (version = 1.4.1). The two-sided Wald test was performed using the Python package NumPy (version = 1.16.4). For assessment of linear correlation, the square of the Pearson coefficient of correlation ${R}^{2}$ was calculated:

$${R}^{2}= {\left(\frac{1}{n{\sigma }_{x}{\sigma }_{y}}\sum \left[\left({x}_{i}-{\mu }_{x}\right)\left({y}_{i}-{\mu }_{y}\right)\right]\right)}^{2}$$

with the mean of the values ${\mu }_{x}$, the mean of the quantiles ${\mu }_{y}$ and the respective standard deviations ${\sigma }_{x}$ and ${\sigma }_{y}$. The Pearson coefficient of correlation was computed using the Python package NumPy (version = 1.16.4).

## Results

### Random forest for MDS prediction

BM samples from 19 healthy donors and 22 MDS patients were measured using RT-DC. During acquisition, RT-DC uses the contour of each cell (red in Fig. 1A) to compute seven morphological features (see Fig. 1A): Area (A), length (L_x_) height (L_y_), Aspect ratio (*γ*), Deformation (D): Inertia Ratio (I), and Porosity (Ω). During analysis, debris particles and chunks of cells were filtered out, by removing events with cell cross-sectional area A < 25 µm^2^ and A > 200 µm^2^. Furthermore, events with porosity Ω > 1.05 were excluded since those events typically correspond to doublets or badly tracked cells. In each measurement, several hundreds of cells were acquired, resulting in a distribution for each feature. To quantify distribution properties, we computed the *mean* and standard deviation (*std*) of each feature for each measurement. Since presence of outliers typically has a strong effect on the value of the *mean* and *std*, we additionally computed the *median* and the median absolute deviation (*mad*) which are more robust to outliers. As *mean*, *median*, *std*, and *mad* are computed for each of the seven available features, each measurement is described by 28 values. Based on these values, we trained a random forest model to discover relations that allow to predict MDS (see Fig. 1A). After optimizing hyper parameters as described in the materials and methods, the random forest reached an out-of-bag (oob) accuracy of 82.9%. Moreover, we performed stratified K-fold cross validation using K = 2,…,20 to split the dataset into training and validation set. The random forest model was trained for each K (using the default hyper parameters), and the resulting validation accuracy, area under the receiver operating characteristic curve (auc), specificity, and sensitivity can be found in Supplementary Table 2. The average validation accuracy across all models trained using K-fold cross validation is 79.6%.

The feature importance values were determined for the model with optimized hyper parameters (oob accuracy of 82.9%). Interestingly, the four most important features (quantified using the Gini impurity index) all describe the width of the distribution (*mad* and *std*) of features describing cell size (A, L_x_, L_y_) as displayed in Fig. 1B. Additionally, we performed an unsupervised principal component analysis (PCA) using the complete dataset. The first principal component (PC1) separates events that correspond to healthy and MDS (Fig. S1A, Supplementary Information). Figure S1B shows the loadings of PC1 and the highest loadings are present for features describing the width if the distribution of cell size which is in accordance with the result of the random forest.

We picked the feature with the highest importance (A_mad_) and trained another random forest. Despite using just a single feature, the resulting model still reached an oob accuracy of 78.0%. Figure 1C shows the decision boundary of the model and the boxplots show A_mad_ for healthy and MDS samples. Additionally, a two-sided t-test was performed, indicating that A_mad_ is significantly different between healthy and MDS (p = 0.001). Even after a Bonferroni-correction, which reduces the significance threshold to 0.0018 (0.05/28 = 0.0018), the p-value of 0.001 would suggest a rejection of the null-hypothesis. For a more intuitive understanding why A_mad_ is different between healthy and MDS, the distributions of Area are visualized for representative measurements in Fig. 1D. The plot indicates that the distribution for healthy samples is wider compared to MDS which corresponds to higher A_mad_. That effect is enhanced due to a more pronounced population of cells in a size region between 25 and 45 µm^2^.

Some of the RT-DC features describe similar properties. As a result, the feature values can be correlated and a split in a decision tree of a random forest could be performed equally well using either of the correlated features. In such a situation, the importance of features can become different for each iteration in K-fold cross validation. This behavior is displayed in Fig. S2A (Supplementary Information), showing the mean and standard deviation (std) of the feature importance, resulting after K-fold cross validation using K = 2,…,20 (corresponds to 209 model training iterations in total). By combining similar features into three groups [cell size (A, L_x_, L_y_), mechanics (γ, D, I), porosity (Ω)] and combining similar metrics into two groups [location (mean and median) and width of distribution (std and mad)], six unique classes of features are created. The feature importance of a class is obtained by adding the importance values of the features in the class. After that, the std is obtained using the class importance values of the 209 models training iterations (see Fig. S2B, Supplementary Information). For better comparison, we computed the coefficient of variation (CV) for each feature and for each class of features. While the average CV is 1.01 for the original features, the average CV reduces to 0.33 after combining the features to classes. These results indicate that the model puts a similar importance to each class of features, independent which data is chosen for the training data. The class of features that describe the width of the area distribution has the highest importance.

### Correlation between cell mechanics and mutational status

**Figure 2:**
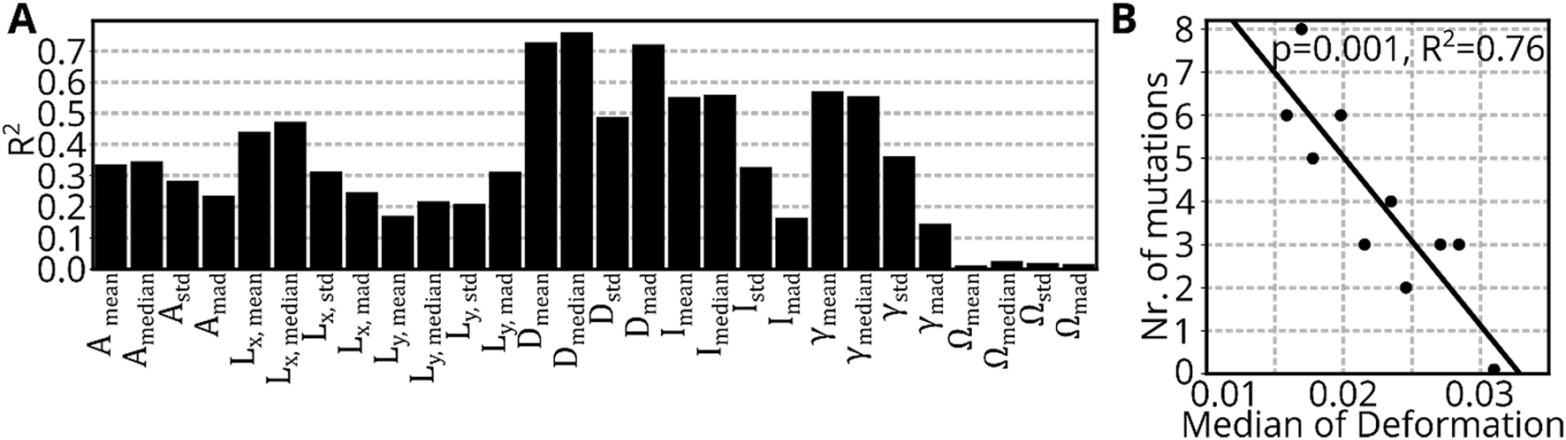
Mechanical properties correlate with number of mutations. (**A**) Barplot shows the square of the Pearson coefficient of correlation (R^2^), quantifying the association of the number of genetic mutations and distribution features from RT-DC. (**B**) Scatterplot shows the number of mutations vs. the median deformation of 10 MDS samples. The line illustrates a linear fit and the corresponding squared Pearson coefficient of correlation is given in the plot.

Targeted sequencing of 54 genes (a list of the tested mutations can be found in Supplementary Table 1) commonly affected in MDS was performed for 10 MDS samples, providing the number of mutations (range, 0–8; *ASXL1*, *KRAS*, *NRAS*, *CEBPA*, *SRSF2*, *CBL*, *TET2*, *ZRSR2*, *EZH2*, *BCOR*, *DNMT3A*, *IDH2*, *RUNX1*, *PTPN11*, *SF3B1*, *WT1*). We computed the square of the Pearson coefficient of correlation ${R}^{2}$ between the number of mutations and each distribution feature of the RT-DC datasets as shown in Fig. 2A. Interestingly, the *mean* and *median* values for features that describe mechanical properties (D, I, *γ*) show a remarkably high negative correlation. The highest correlation is found for the *median* of deformation and the corresponding scatterplot is shown in Fig. 2B. The p-value of 0.001, resulted from a 2-sided Wald test, indicating that the slope of the linear fit is significantly different from zero.

## Discussion

This paper provides a proof-of-concept to use RT-DC for detection of MDS. As RT-DC captures the morphology of cells, the information content is similar to morphology analyses of BM smears which is currently the gold standard for MDS diagnosis. In addition, the mechanical readout of RT-DC is a promising feature as earlier studies showed alterations of the actin cytoskeleton in association with MDS^8–10^.

Current MDS diagnosis routines are under reconsideration due to reproducibility issues, high labor intensiveness, and requirement of expert staff^2,4,19^. These issues could be addressed utilizing a combination of imaging flow cytometry (IFC) for high-throughput acquisition and machine learning for automated data analysis^20,21^. In IFC, fluorescence images are captured which allows labelling of different cell types and intracellular structures. However it was already shown that using deep learning, brightfield images are sufficient for example to predict lineages of differentiation or distinguish cell types in blood^22,23^. Hence, the label-free approach of RT-DC could be advantageous as the staining process can be omitted.

In the present work we employ RT-DC for the first time for detection of MDS. From each captured cell, seven features are computed in real-time, which were then used to train a random forest model, reaching an accuracy of 82.9% for the classification of healthy and MDS samples. As RT-DC performs image analysis in real-time, the MDS classification result could be provided immediately during the measurement. Both, the label-free aspect of RT-DC and the real-time analysis could allow to shorten the time needed for diagnosis.

By employing a model interpretation technique, we found that the width of the distribution of cell sizes is one of the most important criteria used by the random forest classification model. While employing only a single feature for classification lowers the accuracy (78%), it may be more suitable for observation in clinical practice. Interestingly, our finding is in accordance with the WHO guidelines which suggest a consideration of cell sizes during morphology evaluation. Our measurements show consistently that a subpopulation of cells in the size range $25 \mu {\text{m}}^{2}\le A\le 45 \mu {\text{m}}^{2}$ is underrepresented in MDS samples (see Fig. 1D and Supplementary Fig. S3). This effect could be explained by the reduced number of B lymphocyte precursor cells in MDS^24^, which are CD34^+^ and could be present in the sample after CD34 based sorting^25^. Moreover, the histogram of cell sizes in Fig. 1D shows a narrow peak at 50 µm^2^ for MDS, while the healthy counterpart presents a wider distribution. Hence, especially the width of the distribution plays a role, rather than the mean or median which is similar for both samples. However, since only 41 samples have been employed to train and validate the random forest model, the extrapolation of this study on the highly heterogeneous MDS population is limited, as the model could be overfitted to this small dataset. Moreover, random forest models do not perform well in extrapolation tasks. Hence, a larger prospective clinical study is required to reach more decisive conclusions.

Our work considered seven features obtained using RT-DC which can be summarized into three groups: features describing cell size (A, L_x_, L_y_), mechanical properties (*γ*, D, I), and porosity (Ω). However, updated versions of the RT-DC technology are capable to save the brightfield image and compute transparency features in real-time which was shown to allow for discrimination between different blood cell types^26^. Moreover, images can be evaluated by a deep neural net which allows to employ fine grained details of the image for an accurate classification^22,23^. Future research should incorporate those new modalities to improve label-free detection of MDS using RT-DC.

MDS is caused by accumulation of genetic mutations which can be identified by whole genome sequencing. While costs for whole genome sequencing reduced from a hundred million to a thousand dollars during the last 20 years, currently only targeted sequencing plays a role in clinical practice^27^. Here, only chosen genes that are frequently affected in MDS are checked, which is problematic, due to the large genomic heterogeneity present in various types of MDS^28,29^. Therefore, the standard diagnosis relies on an assessment of cell morphology as an indirect readout of genetic properties. Morphological alterations are accompanied by changes in the F-actin distribution and structural changes of the cytoskeleton^8–10^. RT-DC allows to measure mechanical properties of cells that are determined by the cytoskeleton^5,30,31^. It was already shown that diseases like malaria, leukemia, or spherocytosis lead to measurable differences in mechanical properties^26,32^. To link mechanical and genetic changes, we measured HSCs from MDS patients using RT-DC and performed molecular analysis in parallel. Figure 2B indicates that larger numbers of genetic mutations correspond to lower median deformation. Therefore, RT-DC could provide an additional indirect readout of acquired mutations that has low cost per measurement, low measurement time, and offers real-time analysis results. However, despite the high correlation, we would regard this finding as hypothesis-generating due to the small sample size (n = 10). Additionally, we could neither identify an association of mutation type and deformation, nor a significant mechanical difference between the low and the high-risk group (data not shown), but rather the biological features of the blast cells, such as number of mutations, correlated with the mechanical properties. The importance of D_median_ resulting from the random forest model is low (see Fig. 1B). This suggests that D_median_ is similar for healthy and MDS samples. Hence, the approach of correlating D_median_ to infer the number of mutations is only valid for samples for which MDS had already been diagnosed.

HSCs only make up approximately 1% of the cells in the bone marrow^33,34^. To focus our study on this small subpopulation, we used MACS for CD34 enrichment of HSCs prior to the measurement. However, as the cells produced by mutated HSCs are presumably morphologically different from the healthy counterpart, a future endeavor should assess unsorted bone marrow in RT-DC using a similar approach as shown in the present work. Moreover, the efficiency of CD34 isolation is low, which results in small total numbers of cells for the measurement. As a result, our measurements could not fully employ the available throughput-capacity of RT-DC. Samples shown in this manuscript were subjected to cryopreservation and thawing which could potentially alter the cell morphology and MDS prediction outcome. A follow up project should therefore ideally use fresh BM.

Taken together, our study shows that RT-DC has the potential to expand the current status quo of MDS diagnostics. Both, morphological and mechanical readout from RT-DC are promising parameters for identification of MDS. Whether this method can be complementary to the standard diagnostic procedures in the borderline cases or serve as a rapid reliable test in the initial diagnostics remains to be demonstrated in the prospective clinical studies.

## Supplementary Information

Supplementary Information.

## Data Availability

All data shown in this manuscript are publicly available at Zenodo: https://doi.org/10.5281/zenodo.5655848. For each analysis step, a Python script is provided, allowing to reproduce analysis. Execution of the scripts requires a Python environment with packages as stated in the Methods section, or by using PyBox 0.1.0. PyBox is a readily installed Python environment containing all packages at the required version. PyBox is publicly available on GitHub: https://github.com/maikherbig/PyBox.
