# Supplementary material for: Machine learning assisted real-time deformability cytometry of CD34+ cells allows to identify patients with myelodysplastic syndromes
Source: Sci Rep. 2022 Jan 18;12:870. doi: 10.1038/s41598-022-04939-z (PMC8766444; doi:10.1038/s41598-022-04939-z)
Supplement: Supplementary file 1 — Supplementary Information. [file 41598_2022_4939_MOESM1_ESM.docx]

Supplementary Information


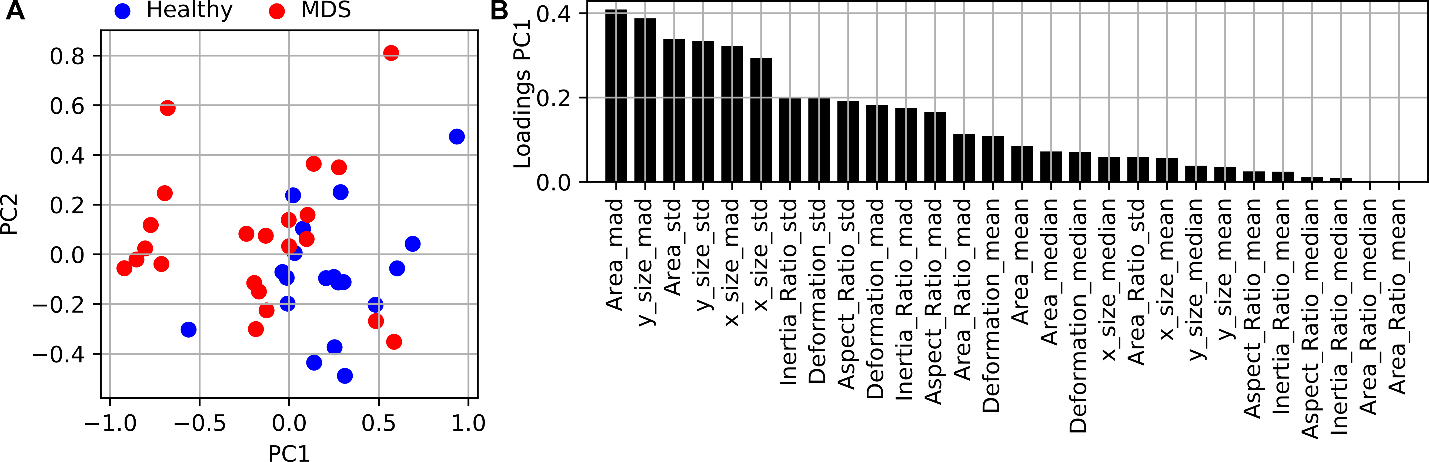


**Supplementary Figure 1: Principal component analysis (PCA).**

An unsupervised PCA was applied to the complete dataset.

(A) The scatterplot shows the resulting principal components 1 and 2.

(B) The barplot shows the loadings of PC1 for each feature.


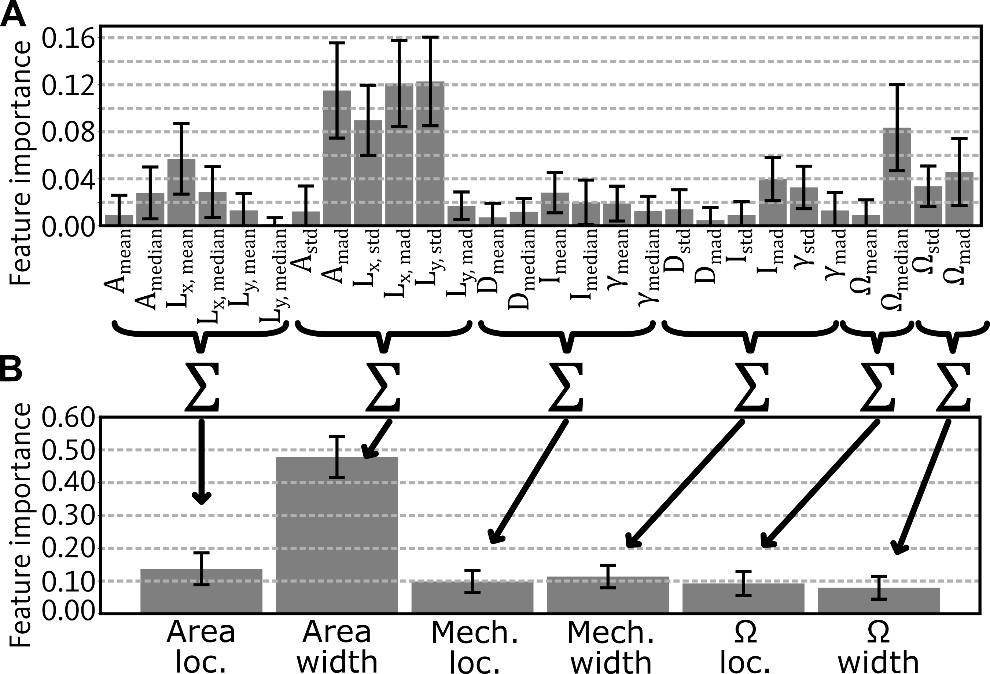


**Supplementary Figure 2: Variance of the feature importance.**

Random forest models were trained using K-fold cross-validation using K=2,…,20, resulting in 209 model training iterations. For each model, the feature importance is obtained.
(A) Barplot shows the mean and std of the feature importance. The average coefficient of variation of for all bars is 1.01.

(B) The sum of the importance values of certain features was computed, resulting in the importance of a class of features. The class importance features of 209 model training iterations were used to compute a mean and std which is shown in the barplot. The average coefficient of variation of for all bars is 0.33.


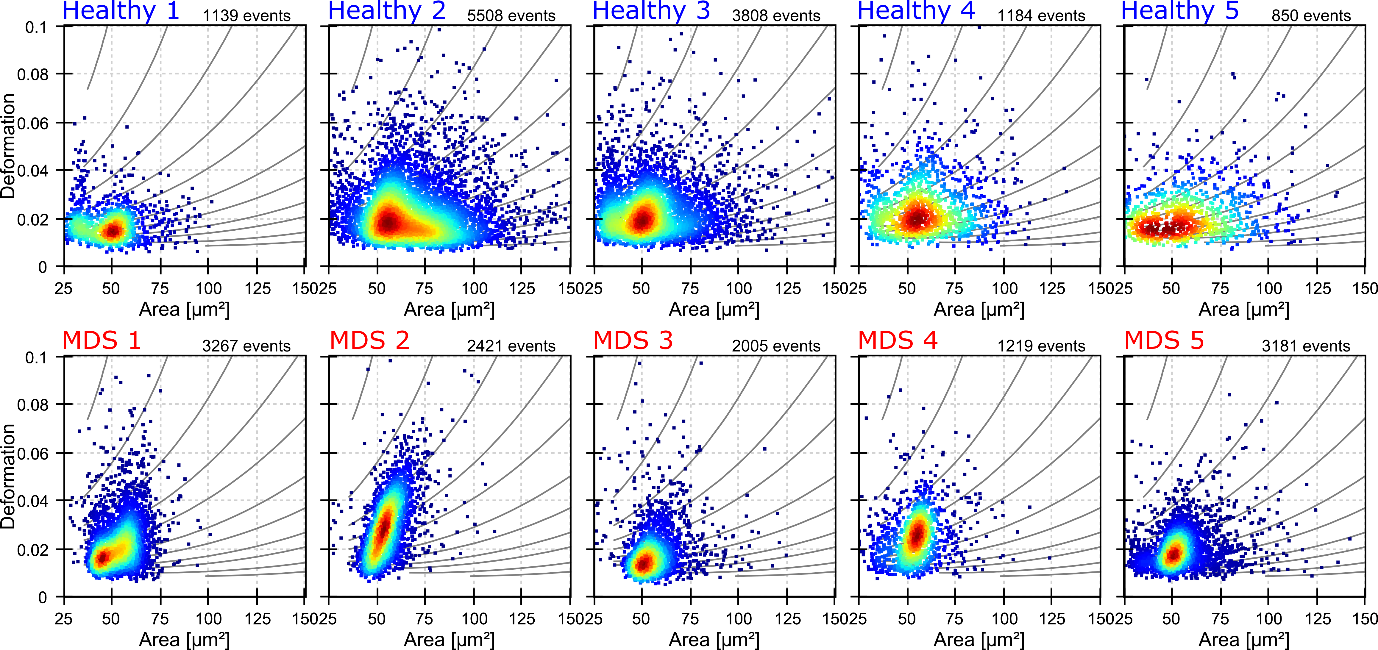


**Supplementary Figure 3: RT-DC measurements of bone marrow samples.**

Scatterplots show measurements of healthy (upper row) and MDS (bottom row) samples. Measurements were performed using a flow rate of 0.04 µl/s and microfluidic chips for RT-DC with a constriction of 20 µm width.

| ABL1 |  | CUX1 |  | JAK2 |  | PTEN |  | WT1 |
| --- | --- | --- | --- | --- | --- | --- | --- | --- |
| ASXL1 |  | DNMT3A |  | JAK3 |  | PTPN11 |  | ZRSR2 |
| ATRX |  | ETV6 |  | KDM6A |  | RAD21 |  |  |
| BCOR |  | EZH2 |  | KIT |  | RUNX1 |  |  |
| BCORL1 |  | FBXW7 |  | KMT2A |  | SETBP1 |  |  |
| BRAF |  | FLT3 |  | KRAS |  | SF3B1 |  |  |
| CALR |  | GATA1 |  | MPL |  | SMC1A |  |  |
| CBL |  | GATA2 |  | MYDD88 |  | SMC3 |  |  |
| CBLB |  | GNAS |  | NOTCH1 |  | SRSF2 |  |  |
| CBLC |  | HRAS |  | NPM1 |  | STAG2 |  |  |
| CDKN2A |  | IDH1 |  | NRAS |  | TET2 |  |  |
| CEBPA |  | IDH2 |  | PDGFRA |  | TP53 |  |  |
| CSF3R |  | IKZF1 |  | PHF6 |  | U2AF1 |  |  |

**Supplementary Table 1: List of tested mutations.**

| Method | Acc. | N_val._ | AUC | Specificity | Sensitivity |
| --- | --- | --- | --- | --- | --- |
| oob | 0.76 | 0 | x | x | x |
| Random 25% of the data as val. set | 0.91 | 11 | 0.95 | 1.00 | 0.86 |
| Stratified 2-fold cross validation | 0.78 | 21 | 0.88 | 0.74 | 0.82 |
| Stratified 3-fold cross validation | 0.76 | 14 | 0.84 | 0.74 | 0.78 |
| Stratified 4-fold cross validation | 0.81 | 10 | 0.87 | 0.79 | 0.83 |
| Stratified 5-fold cross validation | 0.69 | 8 | 0.79 | 0.63 | 0.74 |
| Stratified 6-fold cross validation | 0.81 | 7 | 0.86 | 0.85 | 0.76 |
| Stratified 7-fold cross validation | 0.71 | 6 | 0.91 | 0.64 | 0.79 |
| Stratified 8-fold cross validation | 0.84 | 5 | 0.88 | 0.81 | 0.85 |
| Stratified 9-fold cross validation | 0.81 | 5 | 0.90 | 0.80 | 0.81 |
| Stratified 10-fold cross validation | 0.86 | 4 | 0.89 | 0.90 | 0.85 |
| Stratified 11-fold cross validation | 0.86 | 4 | 0.91 | 0.91 | 0.82 |
| Stratified 12-fold cross validation | 0.83 | 3 | 0.88 | 0.83 | 0.79 |
| Stratified 13-fold cross validation | 0.80 | 3 | 0.88 | 0.77 | 0.81 |
| Stratified 14-fold cross validation | 0.78 | 3 | 0.89 | 0.79 | 0.79 |
| Stratified 15-fold cross validation | 0.79 | 3 | 0.90 | 0.73 | 0.83 |
| Stratified 16-fold cross validation | 0.80 | 3 | 0.89 | 0.72 | 0.84 |
| Stratified 17-fold cross validation | 0.83 | 2 | 0.91 | 0.79 | 0.85 |
| Stratified 18-fold cross validation | 0.82 | 2 | 0.92 | 0.81 | 0.86 |
| Stratified 19-fold cross validation | 0.81 | 2 | 0.89 | 0.79 | 0.84 |
| Stratified 20-fold cross validation | 0.73 | 2 | x | x | 0.78 |

**Supplementary Table 2: Performance values of random forest models.**

The table summarizes the performance of random forest models, trained using different strategies. The columns show the (oob or validation) accuracy (Acc.), the number of samples in the validation set (N_val._), the area under the receiver operating characteristic (AUC), specificity, and sensitivity.
